# Learning-Based Regularization for Cardiac Strain Analysis via Domain Adaptation

**DOI:** 10.1109/TMI.2021.3074033

**Published:** 2021-08-31

**Authors:** Allen Lu, Shawn S. Ahn, Kevinminh Ta, Nripesh Parajuli, John C. Stendahl, Zhao Liu, Nabil E. Boutagy, Geng-Shi Jeng, Lawrence H. Staib, Matthew O’Donnell, Albert J. Sinusas, James S. Duncan

**Affiliations:** Department of Biomedical Engineering, Yale University, New Haven, CT 06511 USA; Department of Biomedical Engineering, Yale University, New Haven, CT 06511 USA; Department of Biomedical Engineering, Yale University, New Haven, CT 06511 USA; Caption Health, San Francisco, CA 94132 USA; Section of Cardiovascular Medicine, Department of Internal Medicine, Yale University, New Haven, CT 06511 USA; Department of Radiology and Biomedical Imaging, Yale University, New Haven, CT 06511 USA; Section of Cardiovascular Medicine, Department of Internal Medicine, Yale University, New Haven, CT 06511 USA; Institute of Electronics, National Yang Ming Chiao Tung University, Hsinchu 30010, Taiwan; Department of Radiology and Biomedical Imaging, Yale University, New Haven, CT 06511 USA, Department of Biomedical Engineering, Yale University, New Haven, CT 06511 USA, Department of Electrical Engineering, Yale University, New Haven, CT 06511 USA; Department of Bioengineering, University of Washington, Seattle, WA 98015 USA; Section of Cardiovascular Medicine, Department of Internal Medicine, Yale University, New Haven, CT 06511 USA, Department of Radiology and Biomedical Imaging, Yale University, New Haven, CT 06511 USA; Department of Radiology and Biomedical Imaging, Yale University, New Haven, CT 06511 USA, Department of Biomedical Engineering, Yale University, New Haven, CT 06511 USA, Department of Electrical Engineering, Yale University, New Haven, CT 06511 USA

**Keywords:** Cardiac function, echocardiography, motion analysis, machine learning

## Abstract

Reliable motion estimation and strain analysis using 3D+ time echocardiography (4DE) for localization and characterization of myocardial injury is valuable for early detection and targeted interventions. However, motion estimation is difficult due to the low-SNR that stems from the inherent image properties of 4DE, and intelligent regularization is critical for producing reliable motion estimates. In this work, we incorporated the notion of domain adaptation into a supervised neural network regularization framework. We first propose a semi-supervised Multi-Layered Perceptron (MLP) network with biomechanical constraints for learning a latent representation that is shown to have more physiologically plausible displacements. We extended this framework to include a supervised loss term on synthetic data and showed the effects of biomechanical constraints on the network’s ability for domain adaptation. We validated the semi-supervised regularization method on in vivo data with implanted sonomicrometers. Finally, we showed the ability of our semi-supervised learning regularization approach to identify infarct regions using estimated regional strain maps with good agreement to manually traced infarct regions from postmortem excised hearts.

## I. Introduction

### A. Motivation

ISCHEMIC Heart Disease (IHD) remains a major problem in the United States. It is characterized by *myocardial ischemia and infarction* (MI) caused by coronary artery narrowing that reduces the blood and oxygen supply. The reduced blood flow leads to left ventricular dysfunction, which may lead to heart failure and/or death. Therefore, a reliable quantitative assessment of regional cardiac function for localization of myocardial ischemia and infarction is valuable for detection and potential interventions. Utilizing imaging modalities such as Cardiac Magnetic Resonance imaging (CMR), nuclear imaging, Computed Tomography (CT), and echocardiography [1], a number of quantitative and semi-quantitative metrics of regional myocardial function have been proposed, including regional ejection fraction, wall thickening, wall motion, and strain. In comparison to other modalities, 4-dimensional echocardiography (4DE) has advantages of cost-effectiveness and a lack of ionizing radiation. In this work, we focus on developing robust methods for estimation of regional myocardial strain for the left ventricle (LV) from 4DE.

Regional myocardial strain estimation requires accurate and reliable motion tracking of the myocardium. Tracking methods typically follow image appearance or image-derived features over the cardiac cycle to produce a dense *Lagrangian displacement field*, where all vectors reference a material point in the end-diastolic (ED) frame. Most previous motion tracking and registration algorithm development efforts implement a compromise between accuracy and smoothness in the regularization of sparse cardiac deformation fields. For example, some algorithms over-smooth for a more global motion estimation (using e.g. registration algorithms) while others lack the ability to do cost-effective and accurate 3D displacement estimation, such as MR tagging. Therefore, intelligent regularization of the dense displacement field is a necessary step for producing cost-effective and more reliable strain analysis, which provides objective evaluation of regional cardiac health that leads to improved ability for diagnosis and targeted therapy. However, addressing this problem from a supervised learning-based approach reveals a domain adaptation problem, as no ground truth labels of displacement vectors exists on the target domain (i.e. in vivo domain). Thus, we approach the problem of domain adaptation by utilizing biomechanical constraints to regularize the cardiac displacement field generated from a range of motion tracking algorithms.

### B. Related Works

#### 1) Cardiac Motion Tracking Algorithms:

Motion tracking algorithms can largely be divided into two categories: intensity-based tracking and feature-based tracking. Intensity-based tracking includes block matching which assumes a consistent speckle pattern across several consecutive frames in the entire echo sequence. For a particular 3D image patch, a search region is defined in the next image frame to find the patch that maximizes similarity to the initial patch. Motion vector is defined from centers of the two blocks. Since each motion vector is estimated independently, the resulting displacement tends to be spatiotemporally noisy. Therefore, post-processing is often performed [2]-[6].

In contrast to block matching, nonrigid registration simultaneously estimates all voxel-wise displacements by deforming an entire image frame to match a subsequent image frame optimally and produces a displacement field represented by smooth kernels such as B-splines, Thin-plate splines, or Radial Basis Functions (RBF). Different parameterizations to characterize cardiac motion and strain include cubic splines [7], B-Spline with 3-D bending energy [8], and LV-shaped coordinate system parameterized with B-splines [9]. Registration-based methods are computationally intensive due to solving for all voxel displacements with global coarse-to-fine optimization [10]. In addition, these methods require careful placement of grid points in the image, as misplaced grid points may bias myocardial motion such as when the grid points cover both the blood pool and the myocardium in the image [11].

Feature-based tracking extracts image features and tracks these features over the image sequence. These features include curvature [12], texture [13] or shapes and surfaces [11], [12], [14]-[17]. However, feature tracking methods have some weaknesses. First, the majority of methods focus on spatial regularization and disregard temporal regularization [12], [17]. Second, feature tracking performance depends on performance of feature extraction, and ultrasound segmentation is a challenging, non-trivial task. Third, cardiac motion may have a torsional component, and rotationally invariant features are difficult to extract. Lastly, feature tracking methods produce sparse displacements only, and postprocess spatial interpolation and regularization step is required to generate dense displacement fields.

#### 2) Regularization Models:

To address the sparsity of displacement field, regularization models are embedded in intensity and feature-based tracking methods that enforce physiologically-plausible motion behavior, such as spatiotemporal smoothness, tissue incompressibility, and temporal periodicity. Free Form Deformation (FFD) models lay a lattice of rectangular set of control points on the image domain [18], [19]. These control points are displaced from their original locations, and the resulting deformation is represented by a set of polynomial basis functions such as B-splines. As a result, the local displacements enclosed within the control points are implicitly regularized. The choice of basis function determines local smoothness which results in an inherent trade-off between smoothness and accuracy. Since improperly placed control points may bias displacement estimation, Extended Free Form Deformation (EFFD) models are designed to overcome issues caused by the rectangular grid from FFD [20]. EFFD models define control point lattices that are adapted to the heart, such as cylindrical or anatomical [7], [9], [21]-[23].

Finite Element Method (FEM) models start by dividing the myocardium using meshing techniques that facilitate incorporation of biomechanical modeling parameters. For example, the authors in [12] imposed a transversely isotropic linear elastic model that incorporated a fiber model that enforced motion in myocardial directions. However, cardiac deformation in ischemic regions was determined to be exponential [24]. Furthermore, although the process of finite element mesh has been fairly easy to personalize in recent years [25], the difficulty in segmenting out the myocardium accurately due to lung artifacts and rib shadowing have made it difficult to use FEM especially in ultrasound. In contrast to FEM, Radial Basis Function (RBF)-based displacement representations do not require explicit mesh construction and are hence referred to as **mesh-free**. Work done in [26] specifically used the computationally advantageous Compactly Supported RBF (CSRBF) for both displacement field representation and integration of shape and speckle tracking for strain analysis. The authors of [27] extended this work to incorporate a sparsity penalty for data-driven selection of RBF centers.

The aforementioned regularization models use model-based constraints that may not adhere to typical cardiac motion patterns. Models alone may be too generic to accommodate different pathological motions of the heart. Furthermore, they impose spatial regularization only, and extension to spatiotemporal regularization is non-trivial. To address the spatiotemporal regularization problem, our group has investigated several approaches. First, we used a dictionary learning approach to learn a sparse representation of displacement maps in order to recover a well-regularized true displacements [28]. Second, we extended the dictionary learning-based regularization to a supervised learning framework with a feedforward neural network for spatiotemporal regularization of noisy displacements. This framework was generalized to various tracking methods. Most recently, we proposed combining complementary tracking methods using a multi-view learning model and showed further improved tracking and strain estimation performance. Finally, we applied the multi-view network to in vivo data and showed plausibility for domain adaptation [29]. Our previous work showed that data-driven models using deep learning strategies provide a way to integrate information generated from a synthetic dataset [29], which is FEM-based, with in vivo data. These efforts help overcome the limitations of model-based methods, which are computationally challenging to use in clinical setting, often cannot easily adapt to abnormal clinical scenarios (e.g. infarction or ischemia), and lack local tuning parameters. Nevertheless, physics and anatomy-based left ventricle models are still useful in informing the overall cardiac deformation and stress during the cardiac cycle. Thus, in order to realize the advantages of physical-based modeling using FEM techniques while also addressing the limitations, we utilize a synthetic dataset which was fully generated from a FEM physical model that has ground truth labels to train our data-driven model as a proof-of-concept framework to show that deep learning-based methods can handle other ground truth data easily. However, addressing the problem of regularization using supervised learning-based approaches leads to domain adaptation problems since there are no ground truth labels of displacement vectors in the target domain (in vivo).

#### 3) Domain Adaptation:

Curating an accurate and well-labeled dataset is a difficult and expensive task, especially in medical imaging, due to the clinical expertise required to annotate a given imaging modality dataset. Segmentation and classification problems in medical imaging have large labeled datasets that are freely or easily available. However, motion tracking datasets, specifically for echocardiography, are scarce due to the rarity of ground truth labels. Previous work addresses this issue by generating synthetic datasets [30], but using synthetic datasets inevitably leads to the problem of domain transfer when solving the motion tracking task in the target domain (i.e. in vivo data).

There has been extensive prior work on domain adaptation and domain transfer learning. The main strategy is to guide the feature learning by minimizing the differences between the source and the target features.

Some have proposed domain adaptation models using autoencoders to find common features between the source and target distributions by training [31]. The most popular approach has been using an adversarial loss to minimize the domain shift, learning the features that are both discriminative and invariant to the change of domains. The aim of these models is to confuse the network through an adversarial objective with a discriminator. References [32], [33] proposed adversarial unsupervised adaptation methods to regularize the learning of the source and target mappings by minimizing the distance between source and target mapping distributions. Reference [34] learns a joint distribution of multi-domain images and enforces weight sharing using Generative Adversarial Networks (GANs) [35]. Reference [36] proposed a reverse domain adaptation method using GAN to make real medical images look more like synthetic images. In contrast to adversarial approaches, others have approached the problem as a data reconstruction by using encoder for source label prediction and decoder for target data reconstruction [37], [38]. Extending from this, we take a related, but new, approach as described in the next section.

### C. Key Contributions

Our previously proposed method required the availability of true displacement for learning, and cross-domain prediction performance was typically poor [29]. Despite the advancements in domain adaptation in deep learning, prior methods focus on classification problems which is difficult to translate into motion tracking problems. Also, the adversarial losses that have been commonly used do not produce stable results due to non-convergence, mode collapse, and diminished gradients. Thus, in order to address the problem of domain adaptation in cardiac motion tracking, we propose to use a biomechanically constrained learning-based framework to regularize the displacement field. The noisy and difficult in vivo echocardiography tracking can be better informed by the synthetic dataset that has a smooth and accurate ground truth displacement fields. In this paper, we fully address the problem of domain adaptation as it relates to our previously proposed neural network-based approach while specifically addressing the limitations in that method. Thus, this work is a substantial expansion of [29], where we make the following contributions:

- Develop a complete approach for supervised regularization based on an MLP design.
- Present a novel semi-supervised neural network framework with biomechanical constraints for displacement regularization and domain adaptation.
- Validate the proposed methods on in vivo data with implanted sonomicrometers.
- Illustrate the promise of proposed method for identifying injury zones using estimated regional strain maps.

## II. Feedforward Neural Network Learning

### A. Tracking Methods for Initial Displacement Estimation

We utilize three different representative methods for producing initial noisy estimates of the displacement field for regularization: Radio-frequency-based Block Matching (RFBM), Flow Network Tracking (FNT), and Nonrigid Registration with Free Form Deformation model (FFD).

#### 1) RF-Based Block Matching:

We utilized the RF-based block matching (**RFBM**) algorithm from [5], [39] as an input to our proposed framework. This method is performed on phase-sensitive radio-frequency (RF) images, which precede the log-compression and envelop detection steps and are complex valued. As a result, additional intensity-level information was retained for tracking in contrast to B-Mode images, which are filtered for enhanced visualization. RFBM performs tracking in the natural spherical ultrasound coordinate system that spans axial (in the direction of ultrasound beam), lateral, and elevational directions using Normalized Cross Correlation (NCC) as the similarity metric. The sub-voxel precision displacement in the axial direction was estimated by finding the zero-crossing of the phase of complex NCC, and a second order polynomial was fitted to the voxel-level displacement field in the elevational and lateral directions. Further details of this method can be found in [6].

#### 2) Flow Network Tracking:

We also utilized Flow Network Tracking (**FNT**) [16] as an input to our proposed framework. First, 3D surfaces of endocardium and epicardium were extracted from B-mode image frames using the segmentation method called Dynamical Appearance Model (DAM) developed by [14]. DAM discriminates class appearance differences at multiple scales by finding sparse representations of image patches for each class (i.e. blood vs. myocardium). The trained dictionaries were updated on-line through the cardiac cycle, leveraging spatiotemporal coherence. Chan-Vese level set functions were fitted to the discriminated classes to produce smooth myocardial surfaces [40]. FNT sampled points from the extracted myocardial surfaces and assigned these points as nodes on a graph, and edges were the potential paths through the graph. FNT then solved for the optimal flow across the graph given the following constraints: 1) Sum of outgoing flow is less than or equal to one. 2) Sum of outgoing flow and incoming flow should be equal. The above problem was solved with Linear Programming. The edge weights were precomputed as a function of Euclidean and feature distances among neighborhood points. The feature distances were learned by training a Siamese network that finds an optimal feature distance between two image patches. Feature distance was minimized when two patches were most similar (based on shape) and maximized when most dissimilar [41]. Details of this algorithm can be found in [16].

#### 3) Nonrigid Registration:

We implemented the nonrigid registration-based method developed by [10] using B-mode images. This method has two components. First, a global affine transformation is found between two images of interest that aligns the moving image to the fixed image frame. The objective function was solved with Limited Memory-Broyden-Fletcher-Goldfarb-Shanno (L-BFGS) [42]. This registration method produced a displacement field between two image frames, and we had two ways of utilizing this method to produce a 4-dimensional Lagrangian displacement field. In the first approach, we first registered adjacent frames to produce an Eulerian displacement field for each image frame. We then converted the Eulerian displacement field to a Lagrangian displacement field by temporally interpolating the displacements over time. We referred to this approach as Frame-to-Frame Registration using FFD model (**FFD FtoF**). In the second approach, we registered every frame in the cardiac sequence to the end-diastole frame. This approach directly produced a 4D Lagrangian displacement without the need for conversion. We referred to this approach as Frame 1-to-Frame Registration using FFD (**FFD 1toF**). We utilized results with both of these registration approaches as part of our overall work.

#### 4) Combining Complementary Tracking Methods:

**Fig. 1.**
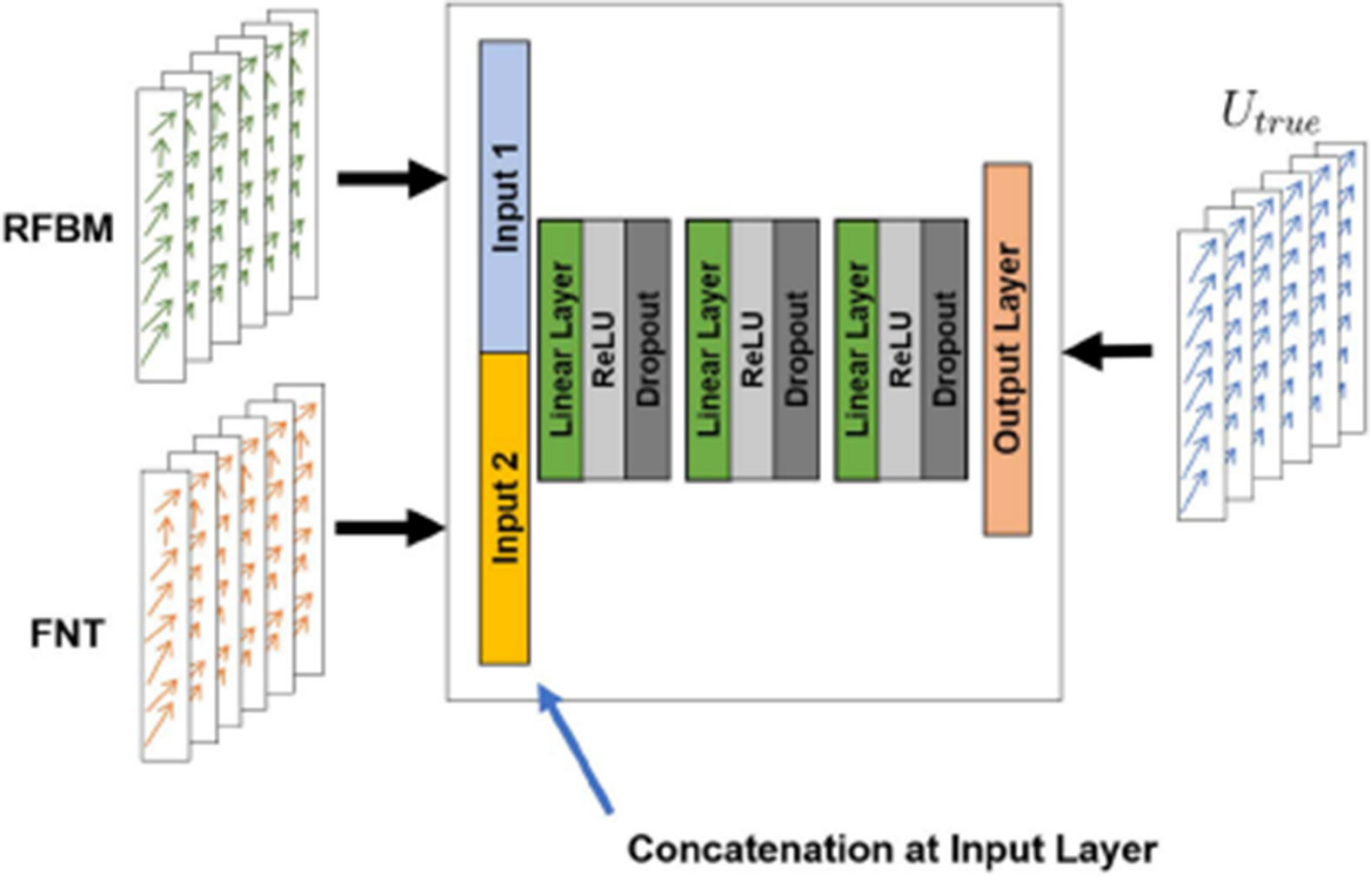
Multi-view learning architecture for integrating RFBM and FNT-generated displacement patches.

**Fig. 2.**
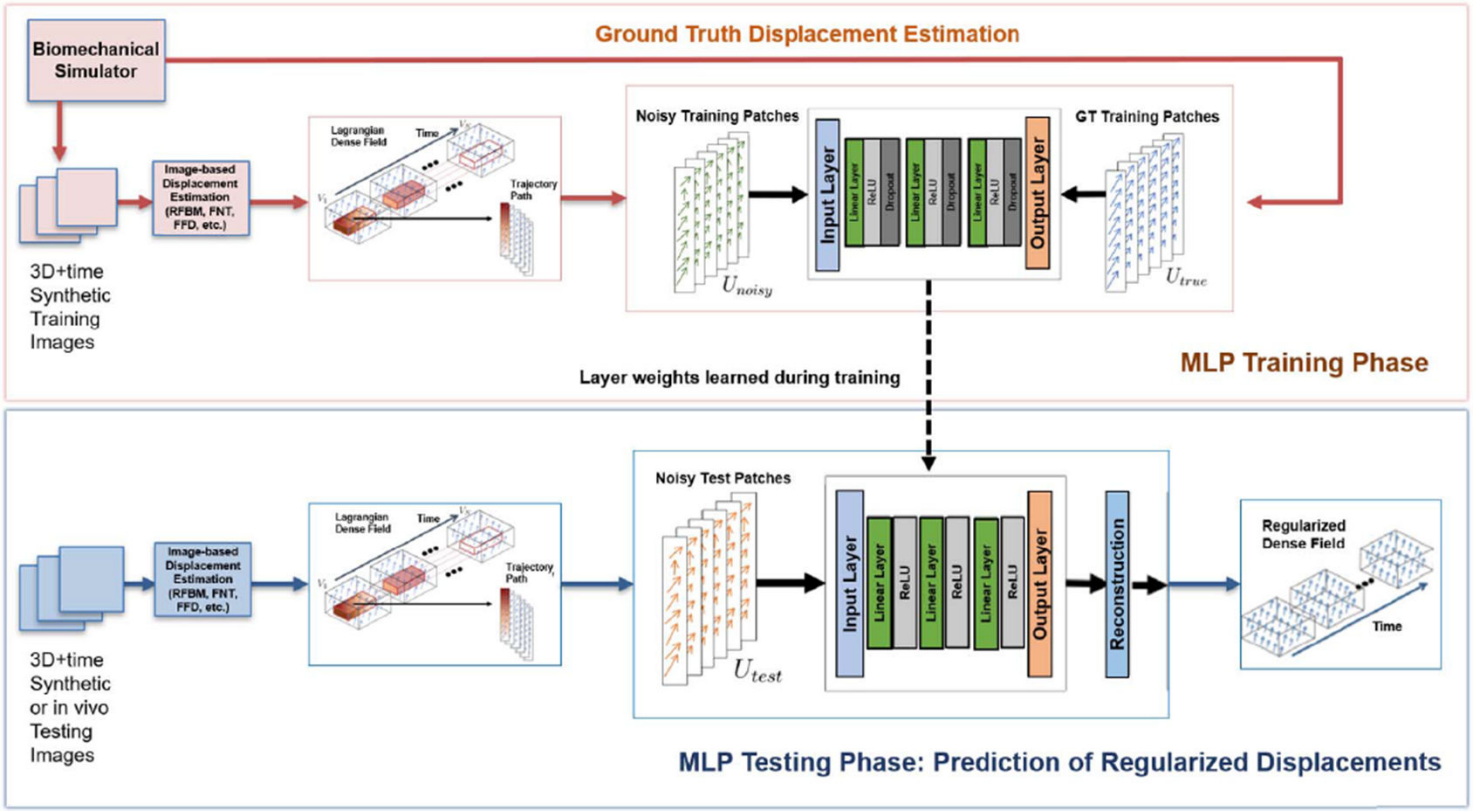
Overview of the method in Section II. The red box indicates the training phase, where we train the MLP using extracted patches and ground truth displacement field. The blue box indicates the testing phase, where we predict the regularized displacement field using the trained MLP.

Each tracking method is unique in capturing certain features in the image that allows it to produce displacement maps. We also aimed to integrate complementary tracking methods applied to inter-modal ultrasound images for overall improved estimations. Our approach was to learn the relationship between complementary tracking methods using multi-view learning. Multi-view learning [43] is a class of machine learning models that combine multiple independent sources of features and has classically been used in the medical imaging community for integrating multiple instances or views of the same object. Inspired by this, we combined the extracted displacement patches from complementary tracking methods at the input layer of our feedforward neural network, and the network produced one set of regularized displacements from both of these sources. As a result, the network implicitly learned to weigh the inputs to produce one set of displacement estimates that captured the complementary nature of the inputs. Fig. 1 illustrates the network architecture. This would replace the architecture in Fig. 2 training phases. We validate our multi-view learning architecture by exploring different combinations of complementary tracking methods. First, we looked at combining RFBM and FNT. RFBM has better performance inside the myocardium but has poor performance near the boundaries due to speckle de-correlation. On the other hand, B-mode based FNT tracks extracted myocardial surfaces and provides more reliable displacement estimation performance near the boundaries. Therefore, RFBM and FNT have complementary tracking features. Second, we study the combination of FFD FtoF and FFD 1toF. FFD FtoF displacement field represents the sequential Eulerian displacement map while FFD 1toF represents a reference-based Lagrangian motion field. Therefore, we pair these two methods to combine the Eulerian and Lagrangian displacement fields. Lastly, we test our model on combining FNT and FFD FtoF. FNT relies on tracking surface points and FFD FtoF tracks intensity information from B-mode. This combination allows us to test combining shape/surface-based and appearance-based B-mode derived information.

### B. Synthetic Data

Our use of synthetic dataset was pivotal in not only validating the performance of our algorithms but providing ground-truth for development of learning-based approaches. The synthetic dataset contained eight 3-D echocardiographic sequences developed by [30]. These synthetic image sequences incorporated realistic ultrasound features that simulated the difficulty in tracking real ultrasound image sequences. The 8 individual sequences from the dataset simulated different physiological conditions, including 1 normal, 4 sequences with occlusions in the proximal (ladprox) and distal (laddist) left anterior descending artery, left circumflex artery (lcx), right coronary artery (rca), and 3 sequences with dilated geometry with 1 synchronous (sync) and 2 dyssynchronous left ventricle activation (lbbb, lbbbsmall). The non-dilated geometry image had image sizes of 224 × 176 × 208 voxels with a voxel size of 0.7 × 0.9 × 0.6 *mm*^3^ with frame rate 23 Hz. The dilated geometry had the same image dimensions as the non-dilated geometry but acquired with a frame rate of 21 Hz. Each image sequence contained 2250 sparse ground-truth trajectories $U_{f}^{sp}$ for interpolation to dense field.

### C. Data Preprocessing

By utilizing a manually segmented myocardium as a region of interest (ROI) guidance for the first frame of the image sequence, we first develop a Lagrangian dense displacement field within the actual myocardium, which is a 4-dimensional vector field, where the displacements at each voxel represent the motion in relation to a material point in the reference point, usually end-diastole in cardiac cycle. Since the heart is a moving 3D object, learning from 4D data is required to avoid out-of-plane motion errors and allows the network to fully capture spatiotemporal motion patterns. Also, properly regularized frame-to-frame (over 3D) displacement estimates are critical for the downstream algorithms and for accurately estimating strain for use in identifying injury zones. Therefore, 4D patches were extracted from the dense field as input to the network. The sparse ground-truth trajectories in our synthetic dataset [30] were spatially interpolated to produce dense ground-truth displacement field. Given sparse trajectories $U_{f}^{sp}$ for image frame *f*, we solved for frame-to-frame ground-truth dense displacement field $U_{f}^{*}$ for frame *f* with the following objective function:

(1) $$w^{*}={\;}_{U_{f}}\|Hw-U_{f}^{sp}{\|}_{2}^{2}+\lambda_{1}\|w{\|}_{1}+\lambda_{2}\|\nabla \cdot U_{f}{\|}_{2}^{2}$$

where *H* defined the radial basis function kernels (RBF), *w* are the optimal weights of RBF, and *λ*_1_ and *λ*_2_ are hyper-parameters for *L*_1_ and divergence-free regularization terms. The resulting frame-to-frame or Eulerian displacement field *U_f_* for all *f* were temporally interpolated with respect to material coordinates of the end-diastole frame and accumulated to produce a Lagrangian displacement field for patch extraction.

In order to learn spatiotemporal patterns, overlapping 4-dimensional patches were extracted from Lagrangian displacement fields as illustrated in Fig. 2. Overlapping 4D patches were used instead of non-overlapping patches in order to upsample more data from a single 4D video clip. The 4D patches were then flattened and concatenated to form the training data. This was applied to both ground-truth Lagrangian displacement field and initial noisy displacement field estimates to form *U_true_* and *U*_*noise*_, respectively. Corresponding pairs of *U*_*noise*_ and *U*_*true*_ patches were fed to the feedforward neural network for learning the regularization function.

### D. Neural Network Architecture: Multi-Layered Perceptron (MLP)

The neural network architecture that we utilized to learn the spatiotemporal patterns between the noisy displacement fields and the ground truth displacement fields was a Multi-Layered Perceptron (MLP). The reason why we utilize MLP over a convolutional neural network (CNN) is that the spatial region of interest (ROI) for regularization is already localized at 5 × 5 × 5 spatial pixels. Therefore, CNN, which typically uses a kernel size of 3 × 3 × 3, would not provide a significant advantage as the potential 3 × 3 × 3 window would nearly encompass the entire ROI.

In a supervised learning manner, a sample of training patches and a sample of ground truth patches are fed into either ends of the MLP so that a latent representation between the two inputs can be learned. For synthetic dataset, our network architecture consists of 3 hidden layers with 1000 nodes per layer along with Dropout with probability of 0.2 and a ReLU activation. For the in vivo acute ischemia dataset, our network architecture consists of 7 hidden layers with 1000 nodes per layer with Dropout with probability of 0.5. We trained for 100 epochs and picked the model with the lowest validation loss. The optimizer used was RMSProp optimizer with an initial learning rate at 1e-5. Fig. 2 shows an example of a 3 hidden layer MLP architecture which was used for the synthetic only training. Details of the loss function used for MLP are described in the following paragraphs.

### E. Spatiotemporal Regularization Learning: Neural Network Loss Function

Our goal was to learn the condition distribution that maps the noisy corrupted displacements *U*_*noise*_ to *U*_*true*_ by minimizing the negative log-likelihood, which is equivalent to the cross-entropy between the data distribution *P_data_* and model distribution *P*_*model*_ [44], defined as:

(2) $$C(\theta )=-E_{U_{noise},U_{true}∼P_{data}}\log\;P_{model}(U_{true}|U_{noise})$$

where *θ* are the parameters of the model. The specific form of *P*_*model*_ determines the loss function. Assuming that *P*_*model*_ has a Gaussian distribution, then the mean squared error (MSE) loss was derived as:

(3) $$C(\theta )=\frac{1}{2}E_{U_{noise},U_{true}∼P_{data}}\|U_{true}-f(U_{noise};\theta ){\|}_{2}^{2}+K$$

where *K* is a function of the variance. While MSE loss may be used, we chose to use a Log-Cosh function, which is a smooth Huber loss function that has *L*_1_ behavior for high loss, and *L*_2_ behavior for small loss [45]. Thus, our objective function for supervised regularization loss was:

(4) $$\theta^{*}={\;}_{\theta }\frac{1}{N}\sum_{i=0}^{N-1}\log\;\text{cosh}\left[U_{true}^{\left(i\right)}-f(U_{noise}^{\left(i\right)};\theta )\right]$$

where *N* is the total number of data samples. In addition to feeding pairs of *U*_*noise*_ and *U*_*true*_ displacement patches to the network model (Fig. 2b), pairs of *U*_*true*_ and *U*_*true*_ displacement patches were also fed to the model for data augmentation. In this way, the model learned to regularize high-error displacement patches and avoid biasing low-error displacement patches via learning the identity function.

### F. Domain Adaptation Using Semi-Supervised Learning With Biomechanical Constraints

**Fig. 3.**
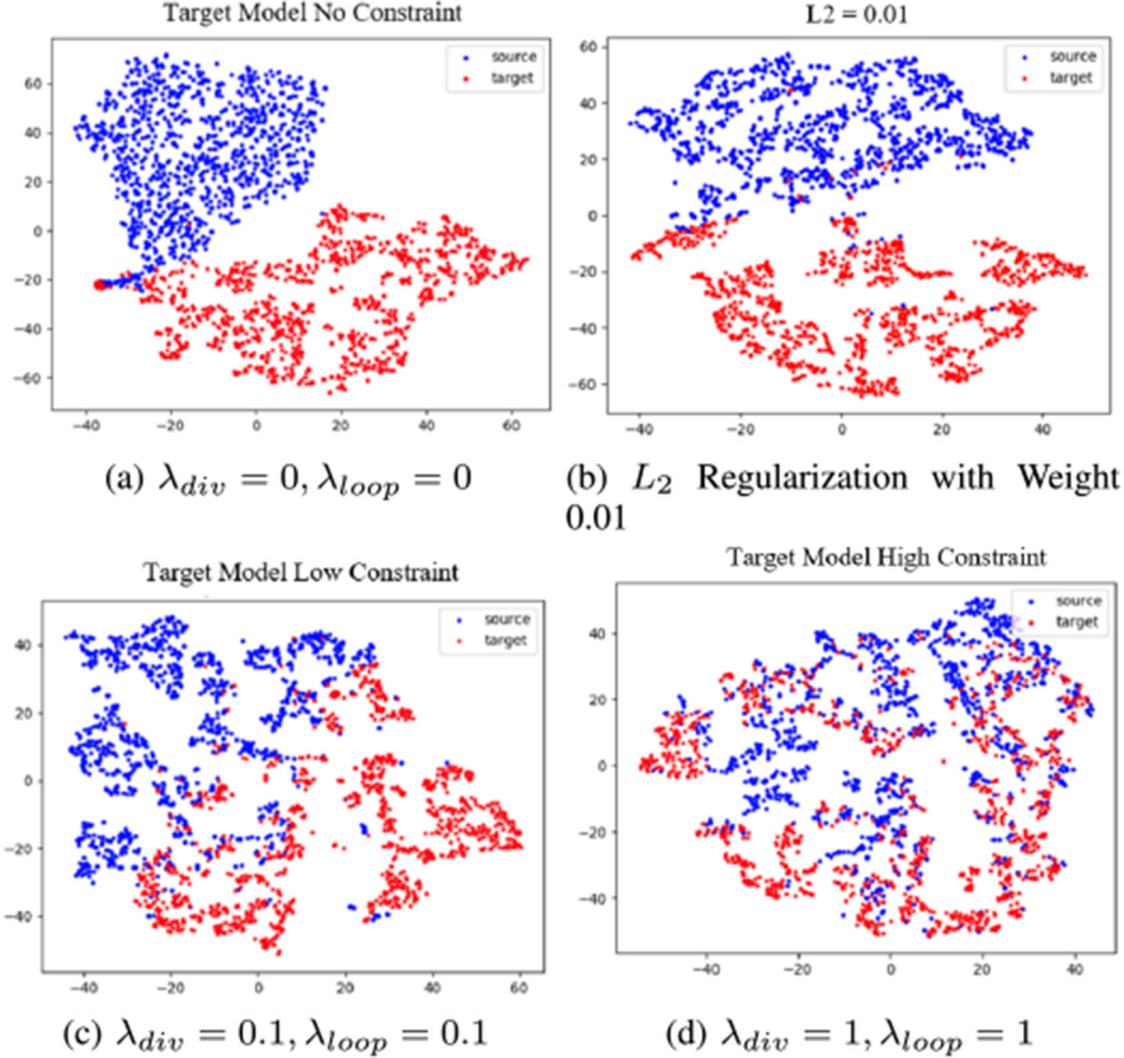
(a) T-SNE plot with no regularization. (b) *L*_2_ Regularization with weight 0.01. (c) t-SNE plot with low regularization. (d) t-SNE plot with high regularization.

Our previously proposed method required the availability of true displacement *U*_*true*_ for learning. This ground-truth is difficult to acquire in practice. Furthermore, training a network with data from one domain (i.e. synthetic domain) and applied on another domain (i.e. in vivo domain) was challenging and usually produced poor results, which can be seen in Fig. 3a. We proposed using a biomechanically-constrained MLP network for learning the latent representation of noisy displacements. MLPs must be constrained in order to learn a useful representation, such as under-complete hidden layers, *L*_1_ penalty on the parameters of the hidden layers, or sparsity constraint on the outputs of hidden layers [44]. Without these constraints, the MLP would simply learn the identity function. In this work, we utilized prior knowledge that well-regularized displacement patches should be biomechanically plausible. Specifically, cardiac tissue deformation is near incompressible, where the volume of myocardial tissue is constant when deformed. In addition, tissue motion is approximately periodic over the cardiac cycle. Leveraging these assumptions, we introduced biomechanically-inspired constraints to the MLP with the objective function in (5):

(5) $$\theta^{*}={\;}_{\theta }\sum_{i}^{N}\{\|U_{noise}^{\left(i\right)}-U_{pred}(\theta {)}^{\left(i\right)}{\|}_{2}^{2}\;\;+\lambda_{div}\sum_{t}^{T}\|(\nabla U_{pred}^{\left(i,t\right)}(\theta )){\|}_{2}^{2}\;\;+\lambda_{loop}\sum_{t}^{T}\|\frac{\partial U_{pred}^{\left(i,t\right)}(\theta )}{\partial t}{\|}_{2}^{2}\}$$

The first term is data adherence between *U*_*noise*_ and predicted displacements *U*_*pred*_ = *f*(*U*_*noise*_; *θ*). The second term enforced incompressibility at each frame *t*, which was measured with *L*_2_ norm of divergence computed as trace of displacement gradient tensor. The third term penalized non-periodicity of cardiac motion. Summation of temporal derivatives over the temporal dimension of perfectly periodic Lagrangian displacements is zero. Thus, we penalized the *L*_2_ norm of temporal derivative of Lagrangian displacements. However, periodicity is not always realistic due to potential probe motion and strong penalization of the term can push the displacement to be near 0. Therefore, we pose periodicity as a *soft* constraint. *λ*_*div*_ and *λ*_*loop*_ were hyperparameters that controlled the influence of divergence and periodicity constraints, respectively. Utilizing these constraints, the MLP was forced to learn a *biomechanically-plausible* representation of noisy Lagrangian displacement patches.

## III. Experiments and Results

### A. Learning Spatiotemporal Regularization Using Synthetic Data

We first quantitatively evaluated the performance of our learning-based regularization on *dense* trajectories (i.e. trajectory for each voxel in the myocardium) to pick out the best tracking algorithm to use for our proposed domain adaptation method. For computational efficiency, we re-sampled each voxel to 0.5 mm^3^ with image size of 75 × 75 × 61 voxels. Training patches were sampled with a stride of 2 in each direction. For normal geometry datasets (**normal, laddist, ladprox, lcx, rca**), our patch sizes were five dimensional: 5×5×5×32×3 for 3 spatial directions, temporal direction, and x-y-z displacement directions. For dilated geometry datasets (**sync, lbbb, lbbbsmall**), our patch sizes were 5×5×5×39×3. In total, we collected around 100,000 patches for training and 22,000 patches for testing. Our network architecture for the synthetic dataset consists of 3 hidden layers with 1000 nodes per layer along with Dropout with probability of 0.2.

Table Ia shows the median tracking error in mm for various different tracking methods. We applied Dictionary Learning-based Regularization (DLR) [28] and Neural Network-based Regularization (NNR) to RFBM, FNT, and FFD FtoF estimates. We imposed a leave-one-out scheme, where we trained on 7 images and tested on the 8th image. Out of the 7 training images, 6 were used for training and 1 was used for validation. The same training scheme was done for both DLR and NNR. The FFD FtoF testing was included in order to compare to an alternative frame to frame approach beyond our own RFBM and FNT methods. We observed that NNR yielded significant improvements in tracking accuracy for all three methods over both initial tracked and dictionary learning-regularized trajectories (DLR).

We further compared our previous efforts based on integration using radial basis functions in [26] with our proposed multi-view network architecture for integration of surface tracking (FNT) and speckle tracking (RFBM) methods, with this method denoted as **RBF-Comb.** in Table I. We used RBF kernels to interpolate the sparse FNT displacements and RFBM displacements, with each sample weighted by a confidence measure. We assumed that FNT was optimal on the myocardial surfaces; thus, we assigned maximum confidence value for all FNT-derived displacements on the surfaces. For RFBM, we used NCC as a confidence measure. We compared the RBF-based combination method with our proposed learning-based integration method, where we input displacement estimates from RFBM and FNT into the multi-view learning framework denoted as **NNR-Comb.**. Furthermore, we noticed that **RBF-Comb.**’s performance was in between the tracking accuracies of FNT and RFBM. Thus, the resulting displacement field estimate was simply an averaging between FNT and RFBM, which resulted in tracking performance that was improved from RFBM but worse than FNT. In comparison, our proposed method **NNR-Comb.** produced better tracking performance than both FNT and RFBM, suggesting that it was effective in leveraging the complementary nature of the two methods. **NNR-Comb.** produced the highest overall tracking accuracy.

We also analyzed our performance via regional strain analysis using a spatial strain map similar to Fig. 6b, where we computed regional strain from dense displacement fields. The computed strain tensors were projected in clinically relevant radial (Rad.), circumferential (Cir.), and longitudinal (Long.) directions. Strain estimation performances were shown in Table Ib. Finally, additional qualitative evaluations of strain curves and maps are shown in [29]. Overall, our learning-based approach to regularize displacements showed a higher accuracy in terms of mean tracking error as well as the strain analysis (Table I) in the synthetic data.

### B. Domain Adaptation With Biomechanical Constraints Using in Vivo Acute Ischemia Model

In order to translate our improved tracking accuracy using learning-based regularization to our in vivo data while also addressing the problem of domain adaptation, we use biomechnical constraints in our regularization terms to bridge between the synthetic data and the in vivo data.

#### 1) Image Acquisition Parameters:

We acquired in vivo 4DE from anesthetized open-chest canines. These canine images were acquired using a Philips iE33 scanner (Philips Medical Systems, Andover, MA) and X7-2 probe and conducted in compliance with Institutional Animal Care and Use Committee policies. We used real time data acquisition sequence ranging from 50-60 frames per second that typically produced around 15-30 3-D volumes for each 4-dimensional sequence.

For each study, we acquired images from the animal under 3 physiological conditions. First, we acquired a baseline image (BL) of the animal. We then introduced a severe stenosis (S) in the mid-left anterior descending (LAD) artery. Finally, we induced a stress condition with a continuous infusion of dobutamine (5*μg/kg/min*) in the continued presence of the severe stenosis (SSDOB). Further details of the experimental set up of the animal studies can be found in [46].

#### 2) Effect of Regularization Terms:

In this section, we analyzed the effect of the biomechanically constrained regularization parameters *λ*_*super*_, *λ*_*loop*_, and *λ*_*div*_ on the performance of domain adaptation. Domain adaptation is the task of improving cross-domain prediction performance. We visualized the effect of these regularization parameters on domain adaptation using t-SNE, which is a nonlinear dimensionality reduction algorithm for visualizing high-dimensional data [47]. It is commonly used for examining the relationship of latent data representations from different domains by clustering them into similar regions that represent the same latent data. We utilize the t-SNE plots to explore the distributions of our synthetic data-derived latent representations and in vivo-derived latent representations. In our experiment, both synthetic displacement patches and in vivo displacement patches were inputted into our semi-supervised learning model, and t-SNE was applied to the output of the *last hidden layer*, which reduced the number of dimensions from 1000 to 2. We plotted the outputs from the hidden layer for four levels of regularization in Fig. 3: part (a) no regularization (*λ*_*div*_ = 0, *λ*_*loop*_ = 0), part (b) *L*_2_ regularization with weight of 0.01, part (c) low regularization (*λ*_*div*_ = 0.1, *λ*_*loop*_ = 0.1), and part (d) high regularization (*λ*_*div*_ = 1, *λ*_*loop*_ = 1). In (a), the hidden layer outputs from the synthetic and in vivo data were completely separated. This indicated that the network implicitly classified the synthetic data (where outputs were true displacements) and in vivo data (where outputs were noisy displacements). As a result, the network predicted in vivo noisy displacements when the input was in vivo noisy displacements, which were not spatially smooth or periodic. This motivated the use of biomechanical regularization to force the predicted in vivo noisy displacements to be spatiotemporally regularized in order to better resemble synthetic displacements, achieving domain adaptation. Thus, we experimented with low regularization and observed a slight “mixing” effect in (c). We further increased regularization and observed a more significant mixing of the outputs of the two domains in (d). This suggested that biomechanical regularization positively influenced the domain adaptation ability of the network model. For general comparison, we included *L*_2_ regularization with weight 0.01 in part (b) to observe how it compares with our biomechanical regularization. This demonstrates that the biomechanical regularization provides better domain adaptation capability compared to other network-based regularization methods such as *L*_2_. One thing to note is that there is a fundamental difference in the *L*_2_ regularization with our biomechanical regularization. *L*_2_ regularization reduces the magnitude of the model parameters. Therefore, as the weights of *L*_2_ regularization gets too high, then the model parameters become smaller in magnitude, which means the model predictions gets smaller in magnitude for both domains. Thus, we might still see a mixing effect if the weights are high enough, but that does not necessarily mean the model is adapted to the data. However, our approach of using biomechanical constraints is that we do not penalize the model parameters but the model output itself, so visually seeing the mixing effect gives us assurance that the model is adapting to the target domain.

#### 3) Sonometric Crystals:

**Fig. 4.**
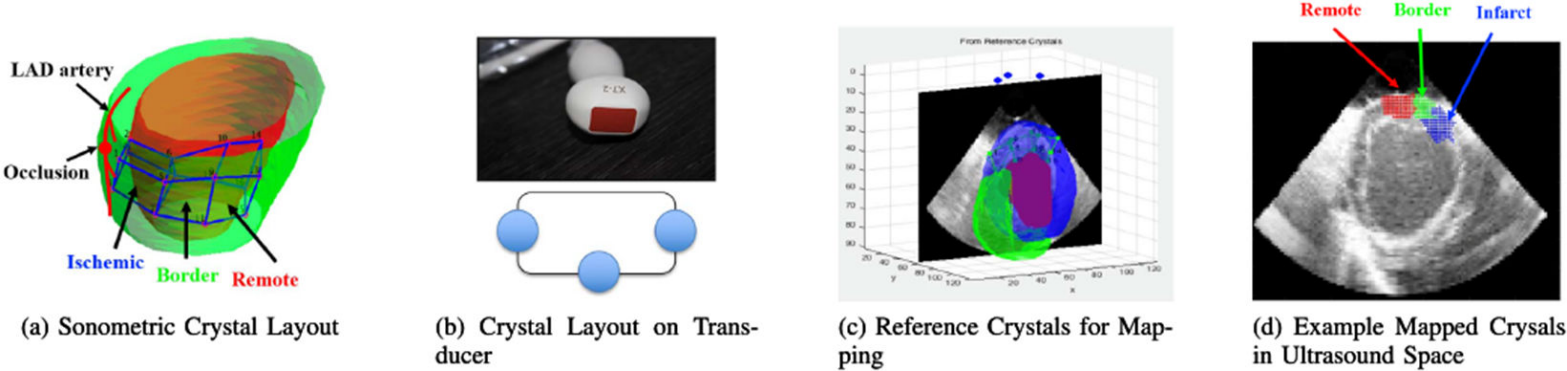
(a) Sonometric crystals layout in relation to LAD artery. (b) Reference crystal on X7-2 transducer arrangement. (c) Mapping crystals onto ultrasound space in 3D. (d) Example crystals mapped on 2D image slice.

We used sonomicrometric transducer crystals with recording instrument and software *Sonosoft* and *Sonoview* (Sonometrics Corporation, London, Ontario, Canada) for recording inter-crystal distances over the cardiac cycle. Cubic arrays with 3 adjacent cubes and 16 total crystals were implanted across the myocardium, where 8 crystals were placed near the endocardial surface, and 8 additional crystals were placed near the epicardial surface. One cube was in the ischemic zone (Ischemic) within the perfusion territory of the stenosed artery. One cube was outside of the ischemic zone (Remote). The last cube was in the middle of the two previously described cubes (Border). These cubes are shown overlaid on sample LV surfaces in Fig. 4a. We computed strain from the 3D cubic array of crystals based on the work in [48]. For each cube, we defined approximately 50 tetrahedral units, or elementary units that consist of 4 out of the possible 8 vertices. A strain tensor was computed for each tetrahedral unit of the cube. Then, we computed the median strain tensors computed from all of the tetrahedral units to yield one final strain tensor. Finally, principal strain was computed via eigen decomposition of the strain tensor.

To utilize the crystal-derived principal strains for validation of strains calculated from in vivo echocardiographic images, we used reference crystals attached to the ultrasound transducer. We made assumptions regarding the locations of those crystals and solved a system of equations for the locations of 16 myocardial crystals. On the X7-2 probe, we attached the reference crystals in the configuration as seen in Fig. 4b. The two crystals facing each other were placed approximately 28 mm apart from each other. The third crystal was approximately 13 mm from the center of the probe surface. We assumed that these crystals were located approximately 5 mm from top of the “ultrasound fan”. Based on these assumptions, we estimated locations of all 3 crystals. Then, for each myocardial crystal, the distances to the reference crystal, computed from crystal positions, should equal to the recorded distances from the crystals. We formulated this relationship as a 3 variable 3 equation problem and solved for the crystal locations. Fig. 4c/d show how the mapping was done and the way it appears on the ultrasound space.

#### 4) Experimental Parameters:

For each dataset, we collected image sequences for three different physiological conditions as mentioned previously: Baseline (BL), Severe Stenosis (S), and Severe Stenosis with Dobutamine (SSDOB). This allowed us to quantify the performance of our algorithm from a clinical perspective, ensuring that we captured the regional (i.e. strain variations across the 3 cubes) and physiological variations.

We computed peak principal strain from each crystal cube and compared with image-derived peak principal strains. Peak strain is the most clinically accepted metric of strain. Therefore, we focus our strain calculation on peak strain. We utilized 4 studies with 3 physiological conditions (BL, S, SSDOB) with 3 cubes (Ischemic, Border, Remote) for each image sequence. In our leave-one-out testing scheme, we have N = 36 samples for comparison. Our testing metric was Pearson correlation computed for the 36 samples. We tested our semi-supervised learning framework on FNT, RFBM, FFD FtoF and FFD 1toF. For each experiment, we extracted approximately 100,000 displacement patches from the synthetic datasets and 100,000 displacement patches from the in vivo datasets, totaling approximately 200,000 patches. To accommodate for the increase in data, we increased the number of hidden layers from 3 (in our synthetic data experiments) to 7 for these in vivo experiments. For each dataset, we computed correlations from peak strains estimated using the initial tracking method (**FFD FtoF, FFD 1toF, RFBM, FNT**). We also showed the computed correlations from peak strains regularized with a neural network model trained *only on the synthetic dataset* (**Synthetic**). Finally, we showed peak strain correlations with our semi-supervised framework (Supervised term and MLP) with biomechanical regularization (**Semi-supervised**), where we set *λ_super_* = 1.

#### 5) Comparison of Methods:

Pearson correlations for the various tracking methods after our proposed domain adaptation method were presented in Table II. RFBM-produced displacements were regularized using *λ*_*div*_ = 0.5 and *λ*_*loop*_ = 0.5. As expected, RFBM without any regularization produced poor results with correlation of 0.01. The severely low correlation stemmed from the fact that principal strain captured the highest deformation and was similar to radial strain, which increased the possibility of over-fitting to noise. A model trained on synthetic data only improved the global correlation to 0.15. However, with using our semi-supervised learning-based regularization, we were able to capture higher correlations of 0.26.

We applied our algorithm to noisy displacement estimates from FNT using *λ*_*div*_ = 0 and *λ*_*loop*_ = 1. We observed an overall increase in global correlations from 0.17 to 0.25. The relatively low correlation for FNT was due to poor segmentation results used for surface tracking. Performance of FNT or any feature tracking-based methods was heavily dependent on the accuracy of the feature extraction process. In this case, FNT relied on segmentation accuracy. In our experiments, we used segmentation method described in [14]. In this method, the end-diastole (ED) frame was manually segmented, and the resulting contours were propagated bi-directionally towards the end-systole (ES) frame. As a result, segmentation errors propagated temporally and were highest at ES frame. This was problematic for computing peak strain, which was typically computed from ED to ES.

We applied our algorithm to noisy displacement estimates from both nonrigid registration approaches. Both of these methods were implemented using *λ*_*div*_ = 0.5 and *λ*_*loop*_ = 0.5. For FFD FtoF, correlation improved from 0.33 to 0.52 for semi-supervised learning approaches. We observed that FFD 1toF produced the highest correlation of 0.6 compared to FFD FtoF, RFBM, and FNT and improved to 0.63 with semi-supervised regularization. FFD 1toF produced higher correlation than FFD FtoF likely due to error of propagation significantly affecting performance of FFD FtoF.

#### 6) Combining Multiple Methods:

Given that our multi-view learning architecture was successful in combining RFBM and FNT displacement patches, we tested integration of other combinations of tracking methods, where we inputted two sets of noisy displacements concatenated at the input layer and produced one set of regularized displacement output. We experimented with two combinations that were thought to be promising from the results seen in Table II. We first combined FNT and FFD FtoF in order to explore the shape/surface-based and appearance based B-mode derive information. FNT relied on tracking surface points, but FFD tracked intensity information. Therefore, these two methods provided independent features and produced overall better performance than the individual methods. Specifically, FNT produced 0.17 correlation, and FFD FtoF produced 0.33 correlation. This combined method produced 0.60 for the semi-supervised models.

We also experimented with utilizing both FFD 1toF and FFD FtoF to see the relationship between different temporal displacement calculations. FFD 1toF registered each frame in the image sequence to 1 reference frame, and in contrast, FFD FtoF registered adjacent frames in the image sequence and converted the resulting Eulerian displacements to Lagrangian displacements. Theoretically, registering adjacent image frames is easier due to smaller deformation between adjacent frames, while registering between a reference image frame (e.g. ED) to another frame in the image cycle (e.g. ES) would be more difficult due to high deformation between the two image frames. On the other hand, the process of converting Eulerian to Lagrangian displacements incurs a propagation of error, but FFD 1toF directly produced Lagrangian displacements that did not require this conversion. Therefore, these two methods were complementary in the temporal domain. Our network combining these two methods produced a correlation of 0.67, which was higher than correlations of 0.33 and 0.60 from the individual methods. All results are listed in Table III.

### C. Strain Analysis Using in Vivo Chronic Infarct Model

To investigate the improved displacement maps generated from our proposed domain adaptation approach, we also evaluate strain in 4D echocardiography which is a derivative of the displacement map and the measurement that is clinically relevant.

Stress echocardiography is useful for detecting regions of ischemia and infarction by observing for wall-motion abnormality. However, rest and stress images are typically analyzed qualitatively, which introduces observer variability. Strain is a useful quantitative metric for localizing the ischemic and infarct regions. Thus, we are investigating utilizing strain as a fully quantitative tool to analyze rest-stress studies in echocardiography. There are two physiological states that are used to analyze ischemic and/or infarct regions by clinicians: rest state and stress state. Differential strain which is the difference between rest and stress strains further gives a better localization of ischemia and infarct that may have been hidden in a single state. Thus, we explore rest strain, stress strain, and differential strain to quantify the degree of ischemic/infarct regions.

We conducted an additional set of studies in dogs with chronic myocardial infarction (N = 4). All studies were performed in compliance with Institutional Animal Care and Use Committee policies. Specifically, a balloon occlusion was introduced in either the Left Anterior Descending (LAD) or the Left Circumflex (LCX) arteries and maintained for 3 hours prior to reflow inducing regional myocardial infarction. This procedure was done on day 0, and the animals were imaged on day 9. For each study, images from two animal states were acquired: rest and stressed with dobutamine (5*μg/kg/min*). For both rest and stress image sequences, peak strains were computed using **FFD 1toF with semi-supervised learning regularization** since FFD 1toF showed the highest Pearson Correlation coefficient for the semi-supervised model (Table II). This initial testing used only the best single method. Future work will look at the combined methods.

Strain maps were calculated using the regularized displacement developed from the acute ischemia model. To compare our strain-derived infarct zones with real infarct zones, we excised the postmortem LV from the animal and cut heart into short axis slices, and manually traced the infarct regions for each LV slice, and reconstructed the 2D slices into a 3D surface. Using this surface, we manually mapped the infarct region onto the ultrasound image.

**Fig. 6.**
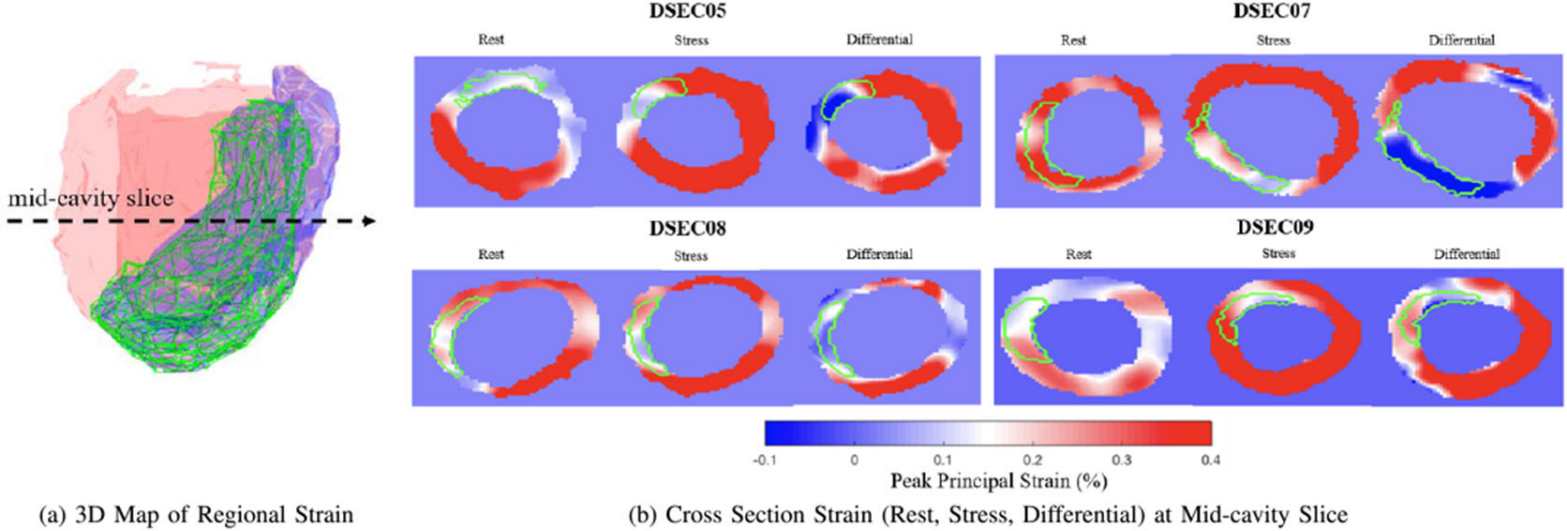
(a) Visualization of 3D map of strain for one chronic study (DSEC05). The dysfunctional area is shown (purple volume) with superimposed infarct area (green mesh). (b) Cross sectional view of rest, stress, and differential strain map at the mid-cavity level for all four chronic animal studies. The color bar indicates the strain value ranges. The red represents the entire myocardium. The blue represents the thresholded infarct zone. The green contour indicates the manually traced infarct zones from the post-mortem excised heart.

Fig. 6A shows a predicted infarct zone using strains from one of the four animal studies. We compare the predicted infarct zone derived from differential strain against the manually traced infarct zone from the post-mortem excised heart. Differential strain was selected since it reveals hidden ischemia that may be imperceptible at either rest or stress states. The predicted infarct zone was found by using a threshold on the principal strain based on visual observation to select the optimum threshold value. Visually, the actual infarct region (green) and the predicted infarct region (blue) show a fairly good match, possibly with some registration error. We also calculated the DICE score between the two. Table IV shows the DICE scores for all four studies. The mean DICE score for the prediction vs. traced infarct was 0.65. This suggests that incorporation of stress imaging and analysis of differential strain improves detection of the infarct zones in the myocardium.

## IV. Discussion

In this work, we expanded our previous work [29] and presented a domain adaptation approach using biomechanically relevant constraints. We also utilized the framework in calculating strain in canine animal studies for ischemic region detector. Also, we noticed RFBM produced high radial strain errors relative to other directions. This was because deformation was highest in the radial direction relative to other directions. Thus, RFBM needed a larger search region to capture high deformation, which meant that it was more likely for RFBM to over-fit to speckle. Overall, strain performance trends in the synthetic data experiments correlated well with the tracking performances of each algorithm. NNR consistently produced improved performance over DLR-produced displacements and all three initial tracking methods.

Also, combining complementary tracking methods to augment the displacement accuracy seems to be consistent across all tracking algorithms, especially when using a neural network approach. This is promising in medical imaging domain such as echocardiography where the image features are noisy and can benefit from accentuating complementary features.

In our attempts at solving the domain adaptation problem, we observed in the in vivo acute ischemia model that the semi-supervised approach using biomechanical constraints to regularize the displacements to be more realistic helped significantly in bridging the synthetic and in vivo domains together. In this case, we brought the in vivo data closer to real or biomechanically meaningful displacements that resemble the synthetic ground truth displacements. We noticed that the Pearson Correlation increased significantly for motion tracking algorithms like RFBM and FNT, while the algorithm FFD did not have as much of an increase. This is because the tracking methods that are not heavily regularized (RFBM, and FNT) stand to benefit more than the already well-regularized methods (FFD). It is also worth noting that the model trained only on synthetic datasets had very low Pearson Correlations. This was likely due to synthetic datasets being significantly different from in vivo dataset in a few aspects. First, synthetic datasets were significantly less noisy compared to the in vivo dataset. Second, the synthetic datasets were generated from human echocardiography images, but our in vivo datasets were acquired from canines. Also, the in vivo experiments represented primarily variations of the left anterior descending (LAD) artery occlusions, while the synthetic datasets were an attempt to model all coronary artery infarctions. Therefore, the pathological perspective of the synthetic dataset is not realistic to that of the in vivo dataset. Third, our synthetic datasets were always oriented vertically in the image domain, but the in vivo datasets were acquired in a variety of probe angles, which resulted in the left ventricle being oriented at different angles. Furthermore, there was only a limited number of synthetically generated echocardiography dataset. This is the current limitation in using the synthetic dataset to model the in vivo displacement results. Nevertheless, data normalization between the two domains with augmentation to the synthetic domain should significantly improve the performance of our semi-supervised regularization approach. However, the advantage of our proposed deep learning-based framework is that the more accurate the training data is, the better the regularization will be. Thus, our work can be used as a framework to guide the displacements from 4D echocardiography by utilizing other higher spatiotemporal or spatial only modality such as human expert delineation and confirmation of a CT cardiac image or MR tagging where there is trusted ground truth displacement vectors. For future work, we will explore ways to move to another modality such as CT and/or MRI to influence spatially dense motion patterns for 4D echocardiography and incorporate a wider variety of pathological cases in our training samples to develop a more robust model.

For the in vivo chronic infarct model, the computed differential strain derived from rest and low dose dobutamine stress images were used to detect regions with infarcted myocardium. This was calculated using a principal strain threshold on the computed differential strain map. The threshold was selected based on the best overlap between predicted infarct and manually traced infarct upon visual inspection. We further tested our method using DICE scores to detect whether definition of the infarct regions using differential strains improved localization of the true infarct region, quantitatively. Some studies (i.e. DSEC07), however, had a relatively low DICE score of 0.55. This was likely due to the misalignment between the rest and stress images during the registration process as well as the accumulation of error from the motion tracking of the left ventricle. Nonetheless, we were able to achieve improved visualizations of infarct region in Fig. 6 as well as a mean DICE of 0.65 among the four studies. In future work, we plan to increase the number of animal studies to better capture the DICE scores between the predicted infarct zone and the actual post-mortem infarct zone and compare them among various tracking methods.

Our domain adaptation approach using learning-based regularization with biomechanical constraints in cardiac displacement map generation is based on the prior knowledge about the physiological movements of the myocardium. Unlike purely computer vision domain adaptation problems, the unique advantage in medical image analysis is that we have a deep understanding of physiology and medicine. Utilizing this information has been shown to be crucial in our attempts at bringing together two different domains like synthetic and in vivo images. Thus, with the added benefit of combining complementary tracking methods, using the prior physiological information about the specific organ system may be key in obtaining better registration results, especially in imaging modalities with low SNR such as echocardiography. Furthermore, regularizing displacement fields allows us to generate a smoother strain map, which is critical to better observe changes that are happening as we move from normal functioning myocardium to infarcted regions.

## V. Conclusion

In this work, we have built a learning-based framework to bridge between imaging-based and model-based estimation of motion fields, proposing a solution that improves imaging-based performance of motion tracking through a regularization based on physics constraints. First, we illustrated the effectiveness of our supervised neural network regularization model on synthetic data, showing improvements in both tracking and strain estimation performance. Furthermore, we proposed a novel semi-supervised MLP network with biomechanical constraints for learning a latent representation that produced more physiologically plausible displacements and extended it to include a supervised loss term on synthetic data and showed the effects of biomechanical constraints on the network’s ability for domain adaptation. We validated the semi-supervised regularization method on in vivo data with implanted sonomicrometers. Finally, we showed, with the semi-supervised learning regularization approach, the ability to identify infarcted regions using estimated regional strain maps with good agreement to manually-traced infarct regions from postmortem excised hearts. This work can further be used to incorporate higher spatiotemporal resolution ground truth data, like MR tagging, to learn accurate and well-regularized displacement fields.

## Figures and Tables

**Fig. 5.**
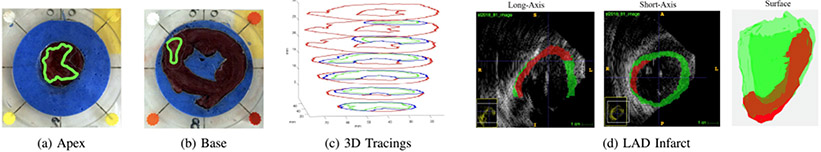
LAD infarct manual tracings from a postmortem excised LV. Part (a) shows a cross-section of the LV near the apex with infarct (green) Part (b) shows a cross-section of the LV near the base with infarct (green). Part (c) shows contours in 3D of traced LV with infarct (green), peri-infarct zones (blue), and myocardium (red). Part (d) shows manually traced LAD infarct (red) onto ultrasound space in the left ventricle (green) and surface map.

**TABLE I:** Median Tracking Error (mm) per Frame Compiled for all 8 Studies for All Trajectories Within Myocardium. Median Strain Error (%) per Frame Between Estimated Strain and Ground-Truth Strain Compiled for All 8 Studies for *all* Trajectories Within Myocardium

Methods	MTE (mm)	Rad.(%)	Cir.(%)	Long.(%)
RFBM	1.64±1.78	21.3±72.6	7.0±44.0	5.9±45.1
RFBM-DLR	1.48±1.55	20.2±33.9	4.9±19.7	5.7±17.5
RFBM-NNR	0.90±0.73	5.9±10.7	2.3±2.6	2.4±3.4
FNT	1.31±0.95	8.1 ±22.0	4.6±12.4	6.1 ±8.7
FNT-DLR	1.28±0.86	8.2±19.2	4.9±10.2	6.0±8.4
FNT-NNR	1.05 ±0.86	4.7±11.4	2.6±3.4	2.6±3.7
FFD FtoF	1.62±1.14	12.3±24.3	4.9±6.0	7.0±16.9
FFD FtoF-DLR	1.61±1.12	12.1±21.7	4.9±5.8	6.9±14.9
FFD FtoF-NNR	1.16±0.80	6.0±10.4	3.0±3.9	3.1±4.1
RBF-Comb.	1.46±0.91	8.5±12.1	3.7±5.3	3.8±5.1
**NNR-Comb.**	**0.82±0.61**	**4.0±9.8**	**1.9±2.2**	**2.2±2.9**

**TABLE II:** Simple vs. Integrated Domain Adaptation: Pearson Correlation (r) Between Crystal and Image-Derived Peak Strains (N = 36). Here, Without Regularization Describes No Neural Network Model, Synthetic Model Describes Domain Adaptation Model Trained Only on Synthetic Data, and Lastly, the Semi-Supervised Study Is the Domain Adaptation Model Incorporating Synthetic Ground Truth With the in Vivo Studies

Studies	RFBM	FNT	FFD FtoF	FFD 1toF
Without Regularization	0.01	0.17	0.33	0.60
Synthetic Model	0.15	0.04	0.37	0.49
Semi-Supervised	**0.26**	**0.25**	**0.52**	**0.63**

**TABLE III:** Crystal vs. Image-Derived Peak Strains: Pearson Correlation (r) Between Crystal and Image-Derived Peak Strains (N = 36) Using Combined Tracking Methods

Studies	Correlation
FNT + FFD FtoF Semi-Supervised	0.60
FFD 1toF + FFD FtoF Semi-Supervised	**0.67**

**TABLE IV:** Dice Score Coefficient Comparison: Manually Traced Infarct From Post-Mortem Data vs. Predicted (4DE-Algorithm-Derived and Thresholded region)

Studies	Traced Infarct vs. Predicted Infarct
DSEC05	0.71
DSEC07	0.55
DSEC08	0.73
DSEC09	0.61
Mean	0.65
Std Dev	0.09
